# No Evidence That Women's Sociosexuality or Self-Perceived Mate Value Predict Their Preferences for Men's Face-Shape Masculinity

**DOI:** 10.1177/14747049251376924

**Published:** 2025-09-08

**Authors:** Pengting Lee, Jingheng Li, Benedict C. Jones, Victor K. M. Shiramizu

**Affiliations:** 1 Department of Psychological Sciences & Health, 3527 University of Strathclyde, UK

**Keywords:** sociosexuality, mate value, facial attractiveness, masculinity, mate preferences

## Abstract

Researchers have suggested that men with more masculine facial characteristics have stronger immune systems but are perceived to be less likely to invest resources in partners and offspring. How women resolve this putative trade-off between the costs and benefits of choosing a masculine mate have previously been reported to be associated with women's openness to uncommitted relationships (i.e., their sociosexuality) and self-perceived mate value. However, not all studies have reported these links and the methods used to assess masculinity preferences in studies reporting these patterns of results (forced-choice tests using stimuli in which masculinity was experimentally manipulated) have recently been criticized for having low ecological validity. Consequently, we tested whether sociosexuality or self-perceived mate value predicted women's masculinity preferences when masculinity preferences were assessed using ratings of individual natural (i.e., unmanipulated) male faces. Our analyses show no evidence that individual differences in women's sociosexuality or self-perceived mate value significantly predicted masculinity preferences. Thus, our results do not support the proposal that sociosexuality and/or self-perceived mate value are important sources of individual differences in women's preferences for male facial masculinity.

## Introduction

Researchers have hypothesized that exaggerated male sex-typical (i.e., masculine) face-shape characteristics in men are associated with traits that are desirable in mates and traits that are undesirable in mates (Little et al., 2001, 2002, 2011; Penton-Voak et al., 1999, 2003). For example, it has been proposed that men displaying masculine face-shape characteristics possess strong immune systems that will be inherited by offspring, meaning that offspring of more masculine men will be healthier (Little et al., 2001, 2002, 2011; Penton-Voak et al., 1999, 2003). However, it has also been proposed that women perceive masculine men to be unlikely to invest time and resources in both their offspring and partners (Little et al., 2001, 2002, 2011; Penton-Voak et al., 1999, 2003; Perrett et al., 1998).

“Trade-off” theory predicts that how women resolve the putative trade-off between the advantages and disadvantages of choosing a masculine mate will differ systematically among women, leading to individual differences in the extent to which women find male faces with masculine shapes attractive (Little et al., 2001, 2002, 2011; Penton-Voak et al., 1999, 2003). For example, trade-off theory predicts that women who are more open to short-term, uncommitted relationships (i.e., women with a less restricted sociosexual orientation) will show stronger preferences for masculine men because the disadvantages of choosing a masculine partner (i.e., low investment) will be less costly in short-term, uncommitted relationships (Little et al., 2001, 2002, 2011; Penton-Voak et al., 1999, 2003). Trade-off theory also predicts that women that perceive themselves to have higher mate value (i.e., women who consider themselves to be particularly attractive) will show stronger preferences for masculine men because women with higher mate value may be better placed to offset the costs of choosing a masculine partner (Docherty et al., 2020; Little et al., 2001, 2011; Penton-Voak et al., 2003). Consistent with these predictions, some studies have reported that women who score higher on measures of openness to short-term, uncommitted relationships or who rate their own attractiveness to be particularly high show stronger preferences for masculine men (Burt et al., 2007; Docherty et al., 2020; Lee et al., 2023; Little et al., 2001; Marcinkowska et al., 2018a, 2021; Sacco et al., 2012; Stower et al., 2020; Waynforth et al., 2005; but see also Alharbi et al., 2021 and Zietsch et al., 2015). However, other recent studies have highlighted two potentially substantial problems with this literature.

First, recent work has cast doubt over the veracity of the two key assumptions that underpin trade-off theory: that men with masculine face shapes are less susceptible to infectious illnesses and are perceived to be unlikely to invest time and resources in their offspring and romantic partners. For example, although some studies have reported that men with more masculine faces report experiencing fewer infectious illnesses (e.g., Thornhill & Gangestad, 2006), other studies, including those that assessed biomarkers of immune response, did not observe significant correlations between men's facial masculinity and susceptibility to infectious illnesses (e.g., Foo et al., 2017). Similarly, while some studies have reported that men with masculine faces were perceived as less likely to be good parents and less likely to be committed to their partners (Boothroyd et al., 2007; Boykin et al., 2023; Kruger, 2006; Perrett et al., 1998), other studies have reported no significant correlations between facial masculinity and perceptions of the likelihood that men will invest effort and other resources in their offspring and partners (Bartlome & Lee, 2023; Dong et al., 2024). Thus, extant research suggests that the proposed links between facial masculinity and both immunocompetence and perceptions of parenting- and relationship-related traits may not be as robust as once appeared to be the case.

Second, recent work has raised concerns about the designs employed in studies reporting that women who score higher on the Revised Sociosexual Orientation Inventory (SOI-R) (i.e., women reporting greater openness to short-term uncommitted relationships) or who rate their own attractiveness to be higher show stronger preferences for masculine men. Studies reporting significant associations between women's sociosexuality and self-rated attractiveness used stimuli in which masculine shape characteristics were experimentally manipulated and presented within forced-choice paradigms. Many researchers have expressed concerns about this method, noting that it lacks ecological validity (Dong et al., 2023; Jones et al., 2023; Jones & Jaeger, 2019; Lee et al., 2021; Lewis, 2017, 2020; Satchell et al., 2023; Scott et al., 2013). Indeed, several recent studies have demonstrated that findings for facial masculinity that were obtained using this method are often not seen when more ecologically valid paradigms are used, such as those in which natural (i.e., unmanipulated) face images are rated individually and shape masculinity measured from the images using facial-metric methods (Dong et al., 2023; Jones et al., 2023; Jones & Jaeger, 2019; Lee et al., 2021; Leger et al., 2023, 2024; Scott et al., 2010).

In light of the above, we tested whether women who scored higher on Penke and Asendorpf's (2008) SOI-R or Edlund and Sagarin's (2014) Mate Value Scale (MVS) showed stronger preferences for masculine shape characteristics in images of men's faces. By contrast with previous studies on this topic, stimuli in the present study were natural (i.e., unmanipulated) images of male faces that were rated individually for attractiveness. Face-shape masculinity was measured from these images using well-established facial metric methods.

## Methods

### Ethics

Procedures were approved by the Department of Psychological Sciences and Health (University of Strathclyde) Ethics Committee, all work was undertaken in accordance with the Declaration of Helsinki, and all participants provided informed consent.

### Participants

Two hundred heterosexual women (mean age = 32.36 years, SD = 7.19 years) took part in this online study and participants were recruited via the prolific participant-recruitment platform. Eligibility criteria for participation were heterosexual women between 18 and 35 years of age who had English as their first language and were resident in the United Kingdom.

### Attractiveness-Rating Task

All participants rated 90 male faces for attractiveness using a 1 (*much less than average*) to 7 (*much more than average*) scale. The 90 images rated were of white male faces (mean age = 27.65 years, SD = 6.00 years), randomly selected from the Chicago Face Database (Ma et al., 2015), and in which the individuals photographed posed with neutral expressions and gaze directed at the camera. The trial order was fully randomized. Inter-rater agreement for these attractiveness ratings was high (Cronbach's alpha = .99) and the mean attractiveness rating was 2.65 (SD = 1.28).

### Questionnaires

In addition to the attractiveness-rating task, all participants completed Penke and Asendorpf's (2008) SOI-R (Cronbach's alpha = .76, *M* = 3.14) and Edlund and Sagarin's (2014) MVS (Cronbach's alpha = .92, *M* = 4.61). Penke and Asendorpf's (2008) SOI-R is a nine-item questionnaire measuring openness to uncommitted sexual relationships. Participants respond to questions such as “With how many different partners have you had sexual intercourse without having an interest in a long-term committed relationship with this person?” using a 9-point scale. Edlund and Sagarin's (2014) MVS is a four-item questionnaire assessing perceptions of own mate value. Participants respond to questions such as “Overall, how would you rate your level of desirability as a partner on the following scale?” using a 7-point scale. Higher scores on these questionnaires indicate greater openness to uncommitted sexual relationships and higher mate value, respectively. Scores on the SOI-R and MVS were not significantly correlated (*r* = .06, *N* = 200, *p* = .41).

### Measuring Face-Shape Masculinity

Face-shape masculinity was objectively assessed for each of the 90 male face images using the facefuns package (Hozleitner & DeBruine, 2021) in R (R Core Team, 2021). This method has been used to assess face-shape masculinity in many previous studies (e.g., Bartlome & Lee, 2023; Cai et al., 2019; Dong et al., 2023; Holzleitner et al., 2019; Komori et al., 2011; Leger et al., 2023). Shape components were first derived from principal component analysis (PCA) of 132 Procrustes-aligned landmark points (see Holzleitner et al., 2019 for a diagram showing these facial landmarks) on each of the 90 male faces. Masculinity scores were then calculated for each image using a vector analysis method (e.g., Bartlome & Lee, 2023; Cai et al., 2019; Dong et al., 2023; Holzleitner et al., 2019; Komori et al., 2011; Leger et al., 2023). This method uses the shape principal components to locate each face on a female-male continuum, defined by calculating the average shape information for the 90 white female faces (mean age = 28.15 years, SD = 5.72 years) in the Chicago Face Database and the average shape information for the 90 male faces presented in the study. Masculinity scores were then derived by projecting each image onto this female-male vector. Higher scores indicate more masculine face shapes. No scores were more than three standard deviations from the mean (i.e., there were no extreme values). The mean face-shape masculinity score was 1.00 (SD = 0.37). Previous work using these stimuli and face-shape masculinity scores reported that shape masculinity scores were significantly and positively correlated with both masculinity and dominance ratings of the faces (Dong et al., 2023).

## Results

All analyses were carried out using R (R Core Team, 2021), with the packages kableExtra 1.3.4 (Zhu, 2021), lme4 (Bates et al., 2015), lmerTest 3.1-3 (Kuznetsova et al., 2017), jtools 2.2.3 (Long, 2022), psych 2.2.5 (Revelle, 2022), and tidyverse 1.3.1 (Wickham, 2021). All data, full outputs, and analysis code are publicly available on the Open Science Framework (https://osf.io/dnvqr/).

**Figure 1.**
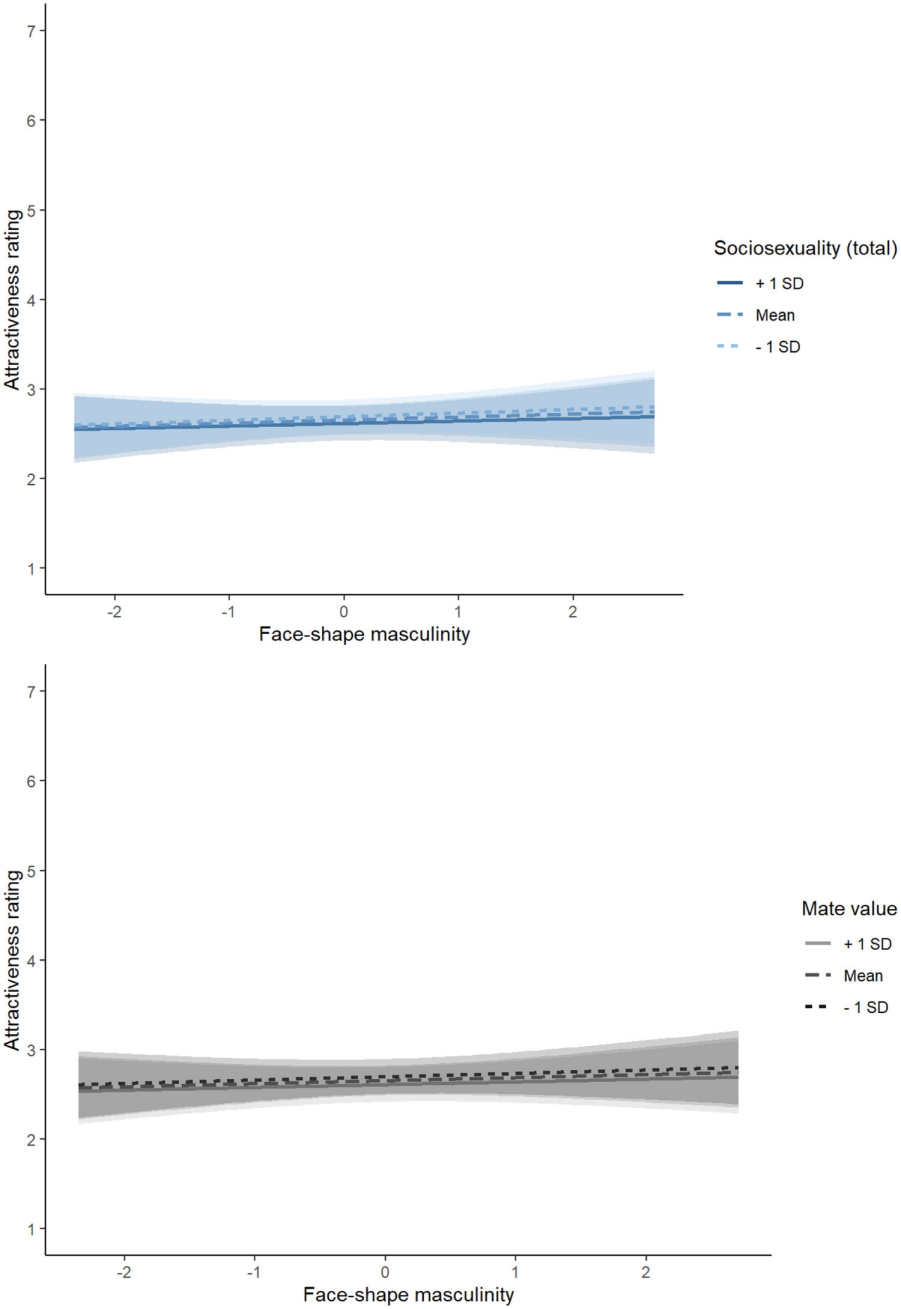
The Interactions Between SOI-R and Face-Shape Masculinity (top Panel) and Between MVS and Face-Shape Masculinity (Bottom Panel).

Abbreviations: SOI-R = revised sociosexual orientation inventory; MVS = Mate Value Scale.

Attractiveness ratings were analyzed using a linear mixed effects model. The model included face-shape masculinity scores, MVS, SOI-R, the two-way interaction between face-shape masculinity scores and MVS, and the two-way interaction between face-shape masculinity scores and SOI-R as predictors. The model also included by-subject random intercepts, by-stimuli random intercepts, by-subject random slopes for masculinity, and by-stimuli random slopes for both MVS and SOI-R. All continuous variables were converted to *z*-scores prior to analyses. Results of this analysis are summarized in Table 1. The effect size (partial Cohen's *d*) for the nonsignificant interaction between shape masculinity and SOI-R was −0.16 (95% CI = −0.58, 0.27) and for the nonsignificant interaction between shape masculinity and MVS was −0.05 (95% CI = −0.47, 0.37). Repeating this analysis, controlling for possible effects of participant age showed the same pattern of results (see https://osf.io/dnvqr/ for full results of this additional analysis). The interactions between SOI-R and face-shape masculinity and between MVS and face-shape masculinity are shown in Figure 1. Repeating our initial analysis, including only SOI-R or MVS (rather than including them both in a single analysis), showed the same pattern of null results (see https://osf.io/dnvqr/ for full results of this additional analysis).

**Table 1.** Results of Linear Mixed Effects Model Testing for Effects of SOI-R (Revised Sociosexual Orientation Inventory) and MVS (Mate Value Scale) on Women's Preferences for Male Face-Shape Masculinity.

	Estimate	Standard error	*t*	*df*	*p*
Intercept	2.655	0.084	31.429	179.031	< .001
MVS	−0.071	0.049	−1.441	206.298	.151
Face-shape masculinity	0.038	0.069	0.542	90.075	.589
SOI-R	−0.036	0.049	−0.728	203.328	.468
MVS × Face-shape masculinity	−0.002	0.009	−0.245	87.910	.807
SOI-R × Face-shape masculinity	−0.006	0.008	−0.716	84.099	.476

The SOI-R can be broken down into three subscales measuring different components of sociosexuality (attitude, desire, and behavior, Penke & Asendorpf, 2008). Some previous studies have suggested that these subscales of the SOI-R have different effects on masculinity preferences and/or that significant effects of sociosexuality that were not evident in analyses of total scores on the SOI-R can be evident when analyzing scores on these subscales (see e.g., Stower et al., 2020). Consequently, we repeated our initial analyses three times, but with the subscales of the SOI-R replacing the total SOI-R (each subscale was analyzed in a separate model). Results of these analyses are reported in full at https://osf.io/dnvqr/ and showed no significant effects. Finally, repeating these analyses, controlling for possible effects of participant age, also showed no significant effects (results of these analyses are also reported in full at https://osf.io/dnvqr/).

Simulations show that the smallest effect we had 80% power to detect (when alpha = .05) for the interaction between sexual dimorphism and sociosexuality was a semi-partial *r* of –.025, and for the interaction between mate value and sexual dimorphism was a semipartial *r* of –.021. This sensitivity analysis (reported in full at https://osf.io/dnvqr/) suggests that our null results are unlikely to be due to insufficient power.

## Discussion

Here we tested for possible associations between women's preferences for men's facial masculinity and both their sociosexuality (assessed using the SOI-R, Penke & Asendorpf, 2008) and self-perceived mate value (assessed using the MVS, Edlund & Sagarin, 2014). Neither the SOI-R nor the MVS predicted the strength of their preferences for male facial masculinity. These null results are inconsistent with previous studies reporting that women who scored higher on the SOI-R or rated themselves to be more attractive showed stronger preferences for masculinity in men's faces (Burt et al., 2007; Docherty et al., 2020; Lee et al., 2023; Little et al., 2001; Marcinkowska et al., 2018a, 2021; Sacco et al., 2012; Stower et al., 2020; Waynforth et al., 2005).

As mentioned in our Introduction section, recent work has highlighted potentially important problems with the methods used in previous work to demonstrate associations between women's masculinity preferences and measures of their sociosexuality or self-rated attractiveness. These studies have generally assessed masculinity preferences using experimentally manipulated stimuli and forced-choice methods, which have been criticized for having poor ecological validity (Dong et al., 2023; Jones et al., 2023; Jones & Jaeger, 2019; Lee et al., 2021; Lewis, 2017, 2020; Satchell et al., 2023; Scott et al., 2013). Our null results add to the growing literature suggesting that findings for facial masculinity obtained using these methods are typically not observed when natural (i.e., unmanipulated) faces are rated individually and face-shape masculinity assessed using facial metric methods (e.g., Dong et al., 2023; Jones et al., 2023; Jones & Jaeger, 2019; Lee et al., 2021; Lewis, 2017, 2020; Scott et al., 2010).

Previous work has suggested that individual differences in women's preferences for masculine men are better explained by genetic factors than by contextual factors, such as sociosexuality or menstrual cycle phase (Zietsch et al., 2015). Recent work has suggested that cultural factors, such as belief in traditional gender-role norms or degree of industrialization, might also influence women's masculinity preferences (e.g., Aksu et al., 2025; Scott et al., 2014). Future work comparing the roles of contextual factors, cultural factors, and genetic factors in individual differences in masculinity preferences may clarify the roles each of these three types of factors play in individual differences in masculinity preferences. Such work might also consider the extent to which these findings generalize across different combinations of stimulus and participant age and ethnicity, as well as generalize to different domains of masculinity preferences (e.g., vocal masculinity, masculinity of body shape, or behavioral displays). Such work might also consider potential moderators not considered in the current study, such as menstrual cycle phase or hormonal contraceptive use. We note here, however, that several recent large-scale studies have found little evidence that these fertility-linked factors influence women's masculinity preferences (e.g., Jones et al., 2018; Marcinkowska et al., 2018b; Stern et al., 2021).

While early work on women's mate preferences predicted that women would show strong preferences for masculine men, findings from many years of subsequent work have suggested that this is not the case (see e.g., Kleisner et al., 2024; Lee et al., 2025). One popular explanation for the absence of strong general preferences for masculine men is that only some women show such preferences (i.e., women who are looking for short-term relationships or who consider themselves to be particularly attractive, see Little et al., 2011, for a review). In the current study, we found no evidence to support these proposals, at least when stimuli were natural (i.e., unmanipulated) faces. Our null results for ratings of unmanipulated faces, together with the significant results reported when masculinity preferences were assessed using manipulated stimuli (e.g., Burt et al., 2007; Docherty et al., 2020; Lee et al., 2023; Little et al., 2001; Marcinkowska et al., 2018a, 2021; Sacco et al., 2012; Stower et al., 2020; Waynforth et al., 2005; but see also Alharbi et al., 2021 and Zietsch et al., 2015) suggest that, while there may be a link between masculinity preferences and sociosexuality and/or self-perceived mate value when manipulated stimuli are used, these results do not occur for assessments of unmanipulated stimuli (i.e., when other facial cues are available to inform attractiveness judgments). If this is the case, it would suggest that effects of sociosexuality and self-perceived mate value on preferences for male facial masculinity are relatively unimportant for real-world partner choices.
